# Evaluating Consistency and Accuracy of GPT-4 Omni to Analyze Thyroid Ultrasound Features and ACR TR Categories to Aid Report Generation

**DOI:** 10.2174/0115734056437835260214191655

**Published:** 2026-03-12

**Authors:** Zebang Yang, Tongyi Huang, Lei Huang, Jiaqian Yao, Lin Jiang, Wenxin Wu, Xiaoyan Xie, Ming Xu, Xiaoer Zhang

**Affiliations:** 1 Department of Medical Ultrasonics, Institution of Diagnostic and Interventional Ultrasound, The First Affiliated Hospital of Sun Yat-sen University, No.58 Zhongshan Er Road, Guangzhou 510080, Guangzhou, People’s Republic of China

**Keywords:** Thyroid nodules, Multimodal large language models, ACR TI-RADS, Gwet agreement coefficient, Images of thyroid nodules, GPT-4 Omni

## Abstract

**Introduction:**

Multimodal large language models, including GPT-4 Omni (GPT-4o), have been applied for facilitating the healthcare process, but their capacity to interpret thyroid sonography images to aid report generation, as well as ways for improvements, are unclear.

**Methods:**

120 thyroid nodules were retrospectively included for evaluation of GPT-4o to analyze ultrasound features and ACR TR categories (version 2017). In a zero-shot setting, 80 original images of unmarked nodules (zero-shot unmarked group) and images with nodules’ boundary artificially depicted by senior radiologists with red circles (zero-shot marked group) were repetitively input into GPT-4o, respectively with identical prompts for 3 attempts without examples. In a few-shot setting, another 40 images with artificially marked nodule boundary (few-shot marked group) were input after 3 examples. The marking gold standard was established by 2 senior radiologists with over 10 years of experience in thyroid sonography. Consistency of GPT-4o was evaluated with the Gwet agreement coefficient (AC1) value calculated. The mean accuracy of GPT-4o across different settings was compared using the Mann-Whitney test with Bonferroni correction, in comparison to the mean accuracy of 2 junior radiologists with 1 and 3 years of experience in thyroid sonography, respectively.

**Results:**

The AC1 values were 0.466 [0.367,0.564], 0.778 [0.696,0.860], 0.823 [0.711,0.934], respectively, for zero-shot unmarked group, zero-shot marked group, and few-shot marked group. The mean accuracy of the 3 groups to judge TR categories was 18.75% [13.78%,23.72%], 42.50% [36.20%,48.80%], 79.17% [71.80%,86.54%]. Zero-shot marked group outperformed zero-shot unmarked group, and the few-shot setting performed even better (*p*<0.001). Particularly, segmentation helped GPT-4o detect composition, shape, and margin of nodules, and a few-shot setting helped detect echogenicity, margin, and calcification (*p*<0.001). Compared with junior radiologists, the few-shot marked group achieved a similar accuracy in identifying composition, echogenicity, calcification, and TR categories (*p*>0.05) and performed even better in identifying the margin of thyroid nodules (*p*=0.004).

**Discussion:**

GPT-4o’s performance to analyze original images of thyroid nodules was insufficient, possibly owing to incorrect nodule recognition and a lack of standardized reference. After adopting segmentation methods and a few-shot setting, its performance was improved significantly.

**Conclusion:**

GPT-4o’s consistency and accuracy of analyzing thyroid sonography images can be gradually improved by segmentation methods and a few-shot setting, and finally achieves a junior-radiologist level in this preliminary study. This can potentially benefit report generation, while multicenter validation is needed.

## 1 INTRODUCTION

Thyroid nodules can be detected in over 60% of the general population, most of which are asymptomatic and clinically insignificant [1]. Imaging modalities, including ultrasound examination, computed tomography (CT), and radionuclide examination, are available for risk stratification and determination of subsequent management, with ultrasound examinations focusing on suspected features recommended as the first-line imaging [2-5]. American College of Radiology Thyroid Imaging Reporting and Data System (ACR TR) criteria (version 2017), a commonly applied classification system emphasizing the risks of hypoechogenicity, taller-than-wide shapes, and microcalcifications of thyroid nodules in clinical practice, has been proved highly sensitive compared to other kinds of TI-RADS, efficient, and better correlated with histological findings with better targeted fine-needle biopsy (FNA) [5-9]. Nevertheless, in regions where less experienced sonographers perform ultrasound examinations, ultrasound features in the ACR TR criteria may not be adequately interpreted. Under these circumstances, preliminary summarized reports generated by artificial intelligence (AI) with corresponding ultrasound findings and ACR TR categories based on images for senior sonographers and radiologists to check, amend, and release reports markedly simplify the ultrasound examination workflow and improve efficiency [5, 10]. Meanwhile, with the integration of rapidly developed AI models in the imaging workflow, robotic AI ultrasound scan further reduces inter-observer discrepancy for the reform of clinical sonography, which needs models with interpretable image findings for the fully automatic examination process [5, 11].

Large language models (LLMs), including Generative Pre-trained Transformer (ChatGPT) by OpenAI, possess remarkable capacity in comprehending and generating human language, showing the potential to facilitate healthcare processes in many ways, including aiding differential diagnosis, supporting scientific reports, and recommending imaging follow-ups for patients [12-20]. In radiology, LLMs have been previously utilized to categorize lesions based on description, generate structured reports from free-text radiologic findings, detect errors or extract data in radiologic reports, and interpret radiologic reports with complicated terminology [21-29]. Recent advancements of multimodal LLMs like GPT-4 with Vision have enabled recognition of images, enabling direct analysis of lesion nature in radiologic images, but the accuracy remains low and unacceptable for clinical practice [30, 31]. Currently, GPT-4 Omni (GPT-4o), a more potent multimodal LLM capable of processing multimedia files, has been developed with interest from medical practitioners [32, 33]. However, to date, research on the capacity of multimodal LLMs to directly analyze real-world thyroid ultrasound images is limited, with only a few studies reporting unsatisfactory accuracyin differentiating benign and malignant nodules with limited clinical translational value [34]. On the one hand, it’s important to find out the potential reasons for the low diagnostic accuracy by investigating the strengths and the weaknesses of multimodal LLMs in sonography analysis so as to explore potential ways to improve them for future clinical application. Recent studies employ various prompting strategies, including the few-shot setting, which greatly increases LLM’s accuracy [30]. A combination of segmentation methods and LLMs enables more effective healthcare applications [35]. But the role of these improvement strategies in LLMs’ direct analysis of medical images remains rarely studied, with limited evidence. On the other hand, given that multimodal LLMs are quite different from deep-learning-based diagnostic models for a single purpose but do much better in interpreting multimedia data in plain or professional language, we hypothesize that integrating current multimodal LLMs at the point of generating preliminary imaging findings from less experienced sonogra-phers or robotic examinations helps improve the ultrasound examination workflow. However, this capacity of GPT-4o has not been thoroughly investigated before. Thus, we aim to investigate the consistency and accuracy of GPT-4o with or without improvement strategies (few-shot training or segmen-tation methods) to analyze ultrasound features and judge the ACR TR category of thyroid nodules, expecting its clinical assistance for report generation and workflow optimization.

## 2 MATERIALS AND METHODS

### 2.1 Study Design

This retrospective study was approved by the institutional review board (Approval No. [20^19^]09), and written informed consent was waived for its retrospective nature. As a preliminary exploration, the present study randomly included 120 de-identified thyroid sonography images from January 2018 to February 2024 in the First Affiliated Hospital of Sun Yat-sen University matching the following criteria: 1) Thyroid nodule was detected in ultrasound scan; 2) Images of both the cross section and the longitudinal section with the maximum diameter of the nodule were clearly preserved; 3) Only a single nodule was present in an image to avoid confounding. Exclusion criteria included poor image quality, lack of images of the cross section or the longitudinal section, and multiple nodules present in an image. No patient information or clinical data was collected.

**Fig. (1):**
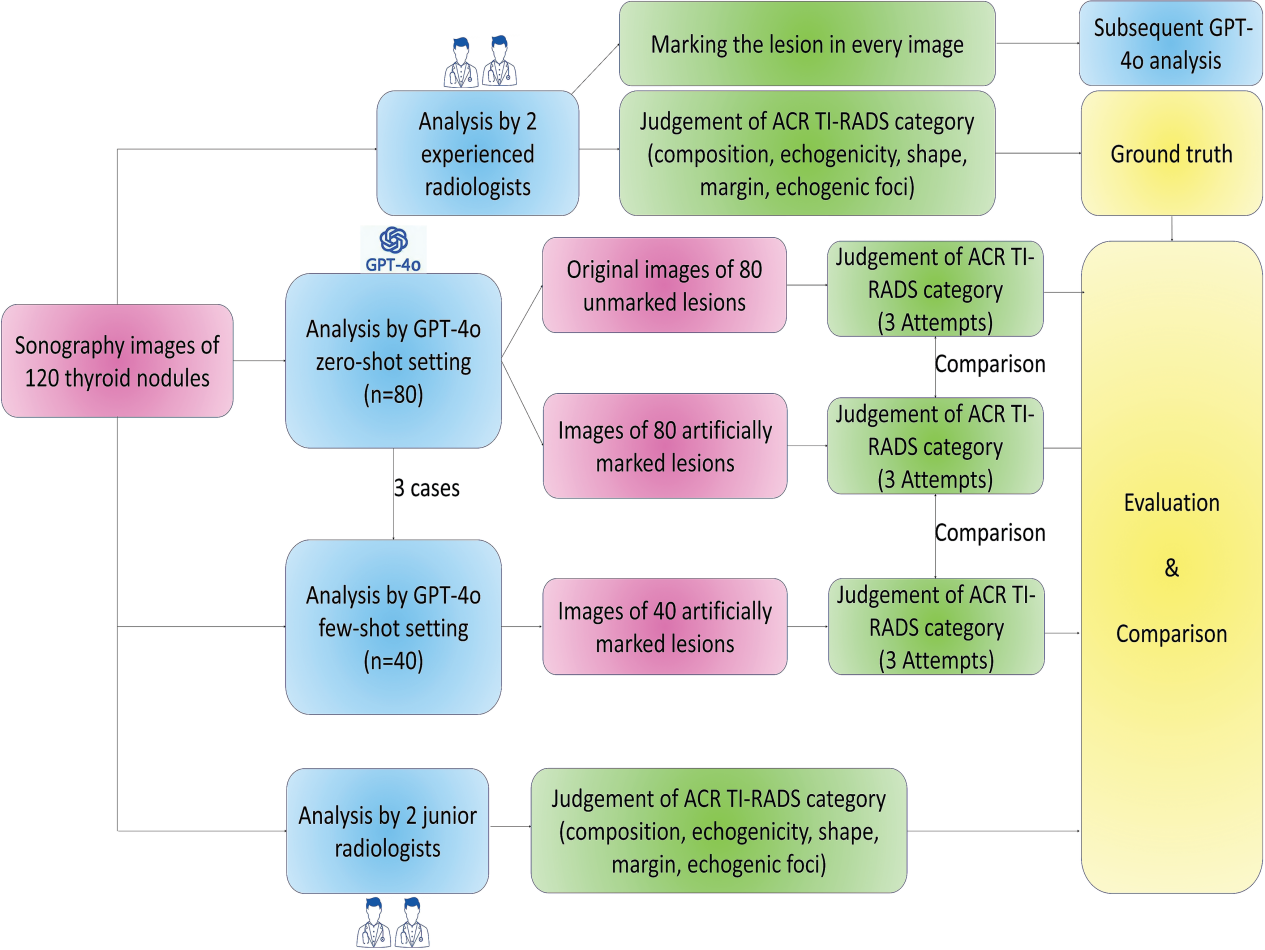
Work flow of the study. 120 sonography images were collected, and the reference standard was established by 2 experienced radiologists after discussion. These images were assigned in a zero-shot setting and a few-shot setting, respectively, and input for evaluation. 2 junior radiologists were asked to analyze the images for comparison.

The sonography images of 120 thyroid nodules were analyzed by 2 senior radiologists with over 10 years of experience in thyroid sonography (the two corresponding authors) who discussed and reached an agreement on the ultrasound features comprising composition, echogenicity, shape, margin, and echogenic foci, together formulating the ACR TR category (version 2017) of each thyroid nodule. They also prepared images of marked nodules for subsequent analysis by artificially depicting the nodule boundaries with thin red circles to simulate segmentation (the red circles were thin around the nodules and would not block nodule characteristics during analysis). Since we attempted to judge GPT-4o’s capacity to recognize subjective visual signs of thyroid nodules instead of benign or malignant nature, the 2 senior radiologists’ consensus on the ultrasound features and corresponding ACR TR categories was set as the gold standard (ground truth). These images were randomly assigned for the zero-shot setting and the few-shot setting of GPT-4o by a ratio of 2:1. In the zero-shot setting of GPT-4o, original images of unmarked lesions (zero-shot unmarked group) and images with nodule boundary artificially marked by 2 senior radiologists (zero-shot marked group) were input, respectively, for evaluation and comparison. To prevent memory bias, 3 cases with different features and TR categories among the 80 nodules were input as examples for the few-shot setting of GPT-4o after the above process was completed. Then, another 40 cases of thyroid nodules marked by the same human experts (few-shot marked group) were input for evaluation (Fig. **1**).

For better interpretation of GPT-4o’s performance, 2 junior radiologists with 1 and 3 years of experience (1 co-first author and the first author) in thyroid sonography were asked to assess the ultrasound features and TR categories of the original unmarked nodules independently, whose accuracy was also calculated and compared with GPT-4o’s average accuracy across settings.

### 2.2 GPT-4 Omni

GPT-4o (OpenAI, USA) was accessed from June 15, 2024, to June 30, 2024, in the study to analyze thyroid nodule images. Only one radiologist operated GPT-4o (the first author) and was carefully supervised and reviewed by 3 senior radiologists (1 co-first author and the 2 corresponding authors). No extra pre-training was performed with radiologic data.

### 2.3 Evaluation of the Performance of Zero-shot Setting of GPT-4o

**Fig. (2):**
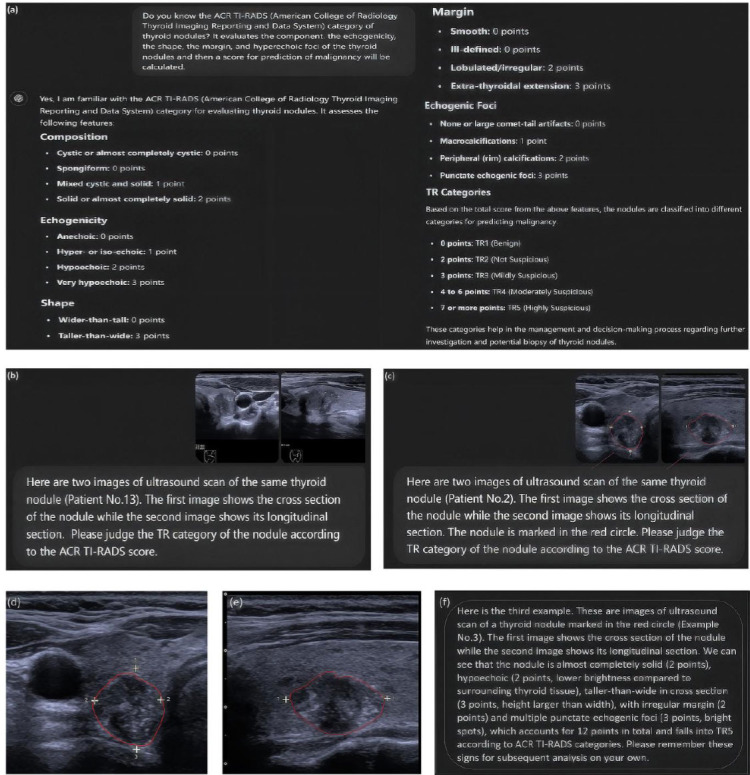
Prompts. (**a**) Introduction prompt input at the beginning of each dialogue. (**b**) Prompt used for original images showing unmarked nodules. (**c**) Prompt used for images with nodules marked by human experts (the red circle in the input images). Compared with the former prompt, GPT-4o was informed that the nodule was marked in the red circle. A detailed format of prompts was shown in the supplementary material. (**d**) The cross-section of an example for few-shot training before another 40 cases were input (Patient No.2, the third example representing TR5 nodules). (**e**) The longitudinal section of an example for few-shot training before another 40 cases were input (Patient No.2, the third example representing TR5 nodules). (**f**) The prompt of this example is for few-shot training. A detailed format of prompts was shown in the supplementary material.

In the zero-shot setting, no example was shown before GPT-4o analyzed images. Beginning with an introduction prompt in the dialogue (Fig. **2a**), sonography images of 80 unmarked thyroid nodules were first input into the GPT-4o model with consistent prompts (Fig. **2b**), and the process was repeated every other day with a new dialogue after all memory was manually deleted to avoid memory bias for 3 rounds (noted as Attempt 1-3). Then, artificially marked images of these 80 nodules were repetitively input and analyzed for 3 attempts by GPT-4o in the same way every other day, with a new dialogue after all memory was manually deleted (Fig. **2c**). The prompts were designed as “Here are two images of an ultrasound scan of the same thyroid nodule (Patient No.X). The first image shows the cross-section of the nodule, while the second image shows its longitudinal section. The nodule is marked in the red circle. Please judge the TR category of the nodule according to the ACR TI-RADS score”. The answers of 5 ACR TI-RADS features, and the TR category of every attempt were collected. Consistency of GPT-4o was evaluated by comparison among attempts in each group, while accuracy was judged by comparison between attempts and the reference standard.

### 2.4 Evaluation of the Performance of the Few-shot Setting of GPT-4o

In the few-shot setting, 3 cases with different features and TR categories were shown to GPT-4o as examples with their reference standard (Fig. **2d**-**f**). Then, sonography images of another 40 marked lesions with the same prompts as the zero-shot marked group (Fig. **2c**) were input into GPT-4o for repetitive 3 attempts every other day and evaluated in the same way as above.

### 2.5 Statistical Analysis

Statistical analysis was performed using SPSS 27.0 software (IBM Co.) and R 4.3.2 software (R Foundation for Statistical Computing). The power of statistical tests mentioned below was estimated using the PASS 21 software (NCSS Co.) to ensure robust statistical interpretations, and the power was estimated to be >0.900 for all tests performed in this study, suggesting promising reliability.

To compare baseline characteristics, Student t test, normality test, and χ^2^ test were applied.

To evaluate the intra-LLM consistency of GPT-4o, the Cohen κ coefficient with 95% confidence interval (95%CI) was used for agreement between every 2 attempts in each group, and the Fleiss κ coefficient with 95%CI was used for agreement among 3 attempts in each group. Given that the ACR TI-RADS multicategory structure may affect the κ coefficient, Gwet agreement coefficient (AC1 value) with 95%CI was also calculated in line with the literature [35]. A κ coefficient or AC1 value of 0.01 to 0.20 suggested slight agreement, 0.21 to 0.40 for fair agreement, 0.41 to 0.60 for moderate agreement, 0.61 to 0.80 for substantial agreement, and 0.81 to 1.00 for almost perfect agreement.

To evaluate the accuracy of GPT-4o, the ratio of correctly recognized signs or correctly categorized nodules to the total number of cases was calculated with 95%CI. The independent accuracy among 3 attempts in each setting was compared using the Kruskal-Wallis test, while the mean accuracy of 3 attempts was compared between the zero-shot unmarked group and the zero-shot marked group, and between the zero-shot marked group and the few-shot marked group using the Mann-Whitney U test, since the accuracy data did not follow a normal distribution. We also calculate the macro-average of its precision, recall, and F1-score of GPT-4o’s analysis of the TR category and 5 ultrasound features.

*p <*0.05 was considered statistically significant. For multiple comparisons, the Bonferroni correction was applied (*p*<0.05/n).

## 3 RESULTS

### 3.1 Baseline Characteristics of Thyroid Nodules

120 cases of thyroid nodules were included, and the reference standard was established. The mean size of thyroid nodules was 1.50±1.14 cm and the proportion of TR1, TR2, TR3, TR4, TR5 categories were 8.33%, 12.50%, 7.5%, 7.5%, 64.17% respectively (Table **1**). No statistically significant difference was found between the 80 images for the zero-shot setting and the 40 images for the few-shot setting (all *p*>0.05).

### 3.2 Intra-LLM Consistency of GPT-4o in the Analysis of Thyroid Sonography

**Fig. (3):**
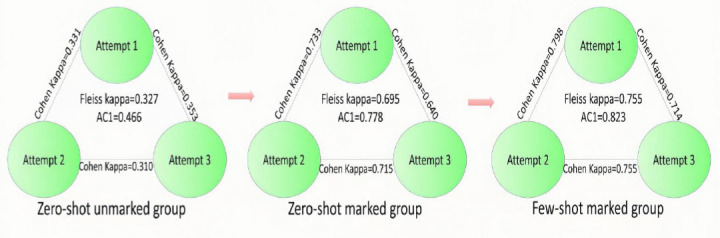
The consistency of GPT-4o to analyze the TR category for 3 groups. The Fleiss κ coefficient and the AC1 value considerably increased after the thyroid nodules were marked and the application of the few-shot setting.

For the zero-shot unmarked group, the Fleiss κ coefficient was 0.327 [0.249,0.406] and the AC1 value was 0.466 [0.367,0.564], indicating a fair to moderate agreement among 3 attempts ofGPT-4owiththe same prompts and images (Table **2**, Fig. **3**). For the zero-shot marked group, the Fleiss κ coefficient was 0.695 [0.608,0.783] and the AC1 value was 0.778 [0.696,0.860], indicating substantial agreement. For the few-shot marked group, the Fleiss κ coefficient was 0.755 [0.651,0.860] and the AC1 value was 0.823 [0.711,0.9^34^], indicating an almost perfect agreement. The Cohen κ coefficients and AC1 values between every two attempts were also measured (Table **2**), suggesting a stable discrepancy of GPT-4o’s answers between every 2 attempts.

**Fig. (4):**
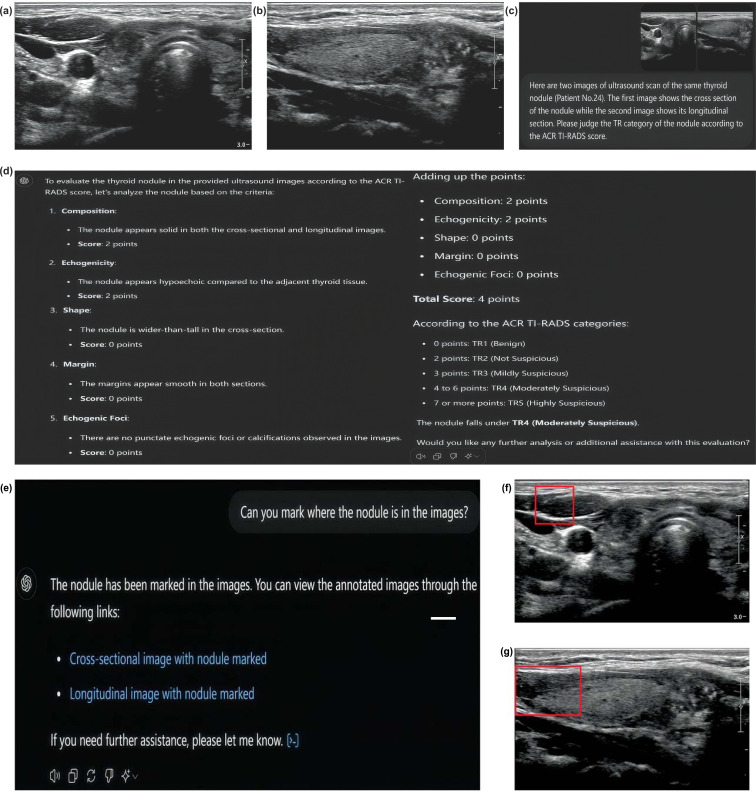
An example of GPT-4o’s answer in a zero-shot unmarked group with a request to mark the nodule. (**a**) Cross-section of the nodule input. (**b**) Longitudinal section of the nodule input. This nodule was solid, hypoechoic, wider-than-tall, lobulated or irregular, with punctate echogenic foci, and fell into the TR5 category. (**c**) Inquiry prompt the same as (**d**) GPT-4o’s analysis of this nodule. GPT-4o generated an incorrect answer of TR4. (**e**) Prompt and answer to the request to mark the nodule. (**f**, **g**) GPT-4o’s marking of the position of the thyroid nodule. Its marking fell outside the thyroid.

To find out the reason for such low consistency among attempts in the zero-shot unmarked group compared with the zero-shot marked group, we tried asking GPT-4o to mark several thyroid nodules in original unmarked images, with mostly incorrect answers obtained (Fig. **4a**-**g**).

### 3.3 Accuracy, Precision, Recall, and F1-score of GPT-4o in Analysis of the TR Category of Thyroid Nodules

**Fig. (5):**
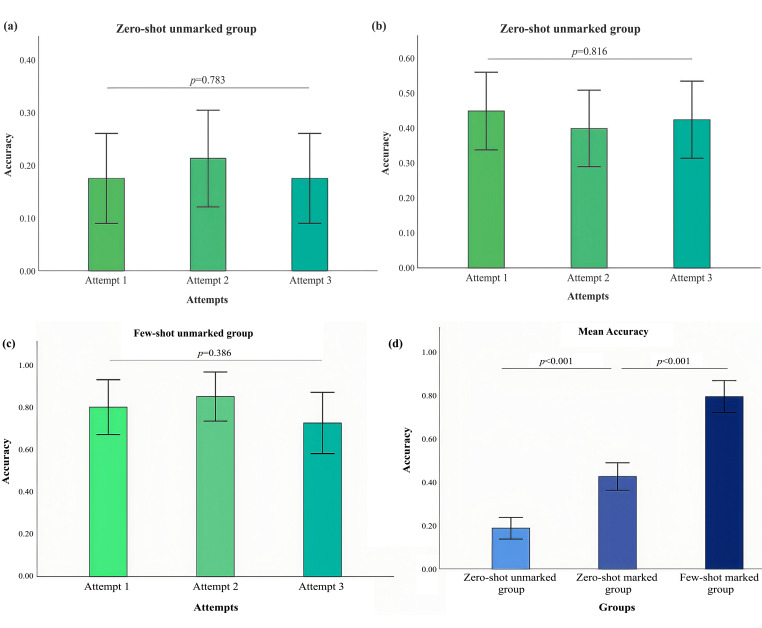
The accuracy of GPT-4o to analyze the TR category for 3 groups. (**a**) Accuracy of GPT-4o in zero-shot unmarked group. No statistically significant difference was found among 3 attempts. The error bar presented the 95%CI of the accuracy. (**b**) Accuracy of GPT-4o in a zero-shot marked group. No statistically significant difference was found among 3 attempts. (**c**) Accuracy of GPT-4o in a few-shot marked group. No statistically significant difference was found among 3 attempts. (**d**) Mean accuracy of GPT-4o to analyze the TR category in zero-shot unmarked group, zero-shot marked group, and the few-shot marked group. A significant difference was observed between every 2 groups.

For the zero-shot unmarked group, the accuracy in categorizing thyroid nodules was 17.50% [8.99%,26.01%], 21.25% [12.09%,30.41%], 17.50% [8.99%,26.01%], respectively, without a statistically significant difference found among 3 attempts (*p*=0.783, Table **3**, Fig. **5a**). For the zero-shot marked group, the accuracy in categorizing thyroid nodules was 45.00% [33.86%,56.14%], 40.00% [29.03%,50.97%], 42.50% [31.43%,53.57%], respectively, without a statistically significant difference found among attempts (*p*=0.816, Fig. **5b**). And for the few-shot unmarked group, the accuracy in categorizing thyroid nodules was 80.00% [67.04%,92.96%], 85.00% [73.43%,96.57%], 72.50% [58.04%,86.96%], respectively, without a statistically significant difference found (*p*=0.386, Table **3**, Fig. **5c**). The mean accuracy of the 3 groups was 18.75% [13.78%,23.72%], 42.50% [36.20%,48.80%], 79.17% [71.80%,86.54%], respectively. The macro-average precision of the 3 groups was 0.264, 0.453, 0.638, while the macro-average recall was 0.209, 0.392, 0.617, respectively. And the macro-average of F1-score was 0.233, 0.420, 0.627 (Table **4**). Significantly higher accuracy was found in the zero-shot marked group than in the zero-shot marked group and in the few-shot marked group than in the zero-shot marked group (both *p*<0.001 with Bonferroni correction, Fig. **5d**).

### 3.4 Accuracy, Precision, Recall, and F1-score of GPT-4o in Analysis of Ultrasound Features of Thyroid Nodules

**Fig. (6):**
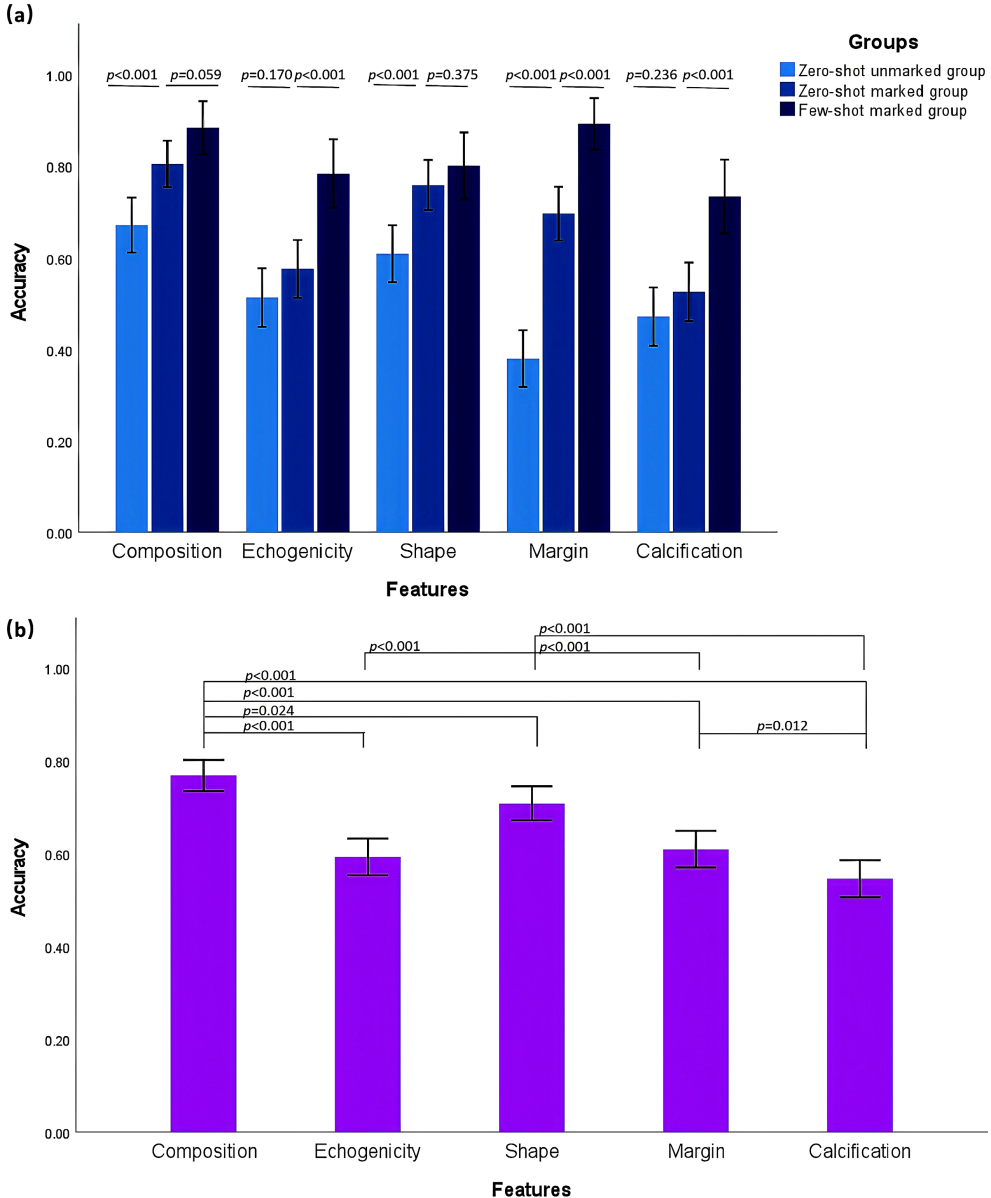
The accuracy of GPT-4o to analyze ACR TI-RADS ultrasound features. (**a**) Accuracy of GPT-4o to analyze ACR TI-RADS ultrasound features among 3 groups. (**b**) Overall accuracy of GPT-4o to analyze 5 ACR TI-RADS ultrasound features. GPT-4o performed better in detecting composition than echogenicity, margin, and echogenic foci (all *p*<0.010 using Bonferroni correction). Also, it performed better in detecting shape than echogenicity, margin, and echogenic foci (all *p*<0.010 using Bonferroni correction).

The mean accuracy of GPT-4o to detect the composition of thyroid nodules was 67.08% [61.10%,73.07%], 80.42% [75.36%,85.47%], 88.33% [82.51%,94.16%] for the zero-shot unmarked group, the zero-shot marked group, and the few-shot marked group. The mean accuracy of GPT-4o to detect echogenicity was 51.25% [44.88%,57.62%], 57.50% [51.20%,63.80%], 78.33% [70.86%,85.81%], respectively. The mean accuracy of GPT-4o to detect shape was 60.83% [54.61%,67.05%], 75.83% [70.38%,81.29%], 80.00% [72.74%,87.26%], respectively. Its mean accuracy to detect margin was 37.92% [31.73%,44.10%], 69.58% [63.72%,75.45%], 89.17% [83.53%,94.81%]. And its mean accuracy to detect echogenic foci was 47.08% [40.72%,53.44%], 52.50% [46.14%,58.86%], 73.33% [65.31%,81.36%] (Table **5**, Fig. **6a**). The precision, recall, and F1-score were also listed in Table **4**. Compared with unmarked nodules in images, the zero-shot marked group performed significantly better in detecting composition, shape, and margin of nodules (all *p*<0.001 with Bonferroni correction). Compared with the zero-shot setting, the few-shot marked group performed significantly better in detecting echogenicity, margin, and echogenic foci (all *p*<0.001 with Bonferroni correction, Table **5**, Fig. **6a**).

Then we analyzed the overall accuracy of GPT-4o to detect each ultrasound feature. GPT-4o showed significantly higher accuracy in recognizing the composition of nodules than echogenicity, margin, and echogenic foci (all *p*<0.001 with Bonferroni correction) and in recognizing the shape of nodules than the remaining 3 features (all *p*<0.001 with Bonferroni correction, Fig. **6b**).

### 3.5 Comparison of Performance between GPT-4o and Junior Radiologists

In this study, the 2 radiologists with 1-year and 3-year experience in thyroid US achieved a mean accuracy of 92.92% [89.65%,96.19%], 76.25% [70.83,81.67%], 92.92% [89.65%, 96.19%], 76.25% [70.83%,81.67%], 70.42% [64.60%,76.23%], 76.25% [70.83%,81.67%] to analyze the composition, echogenicity, shape, margin, echogenic foci, and TR categories of thyroid nodules compared with the gold standard established by senior radiologists, which significantly outperformed zero-shot unmarked group and zero-shot marked group (all *p*<0.001). Compared with less experienced radiologists, GPT-4o achieved a comparable mean accuracy to detect composition (*p*=0.144), echogenicity (*p*=0.659), echogenic foci (*p*=0.564), and TR categories (*p*=0.534) and a significantly higher mean accuracy to identify the margin (*p*=0.004), but junior radiologists performed better to identify the shape of nodules (*p*<0.001).

## 4 DISCUSSION

The current study investigated the performance of GPT-4o to interpret detailed ultrasound features of thyroid nodules under different settings for an AI-assisted ultrasound workflow. Consistency and accuracy of GPT-4o’s judgment towards original thyroid nodule images were insufficient, but they were considerably increased by the segmentation method and the few-shot setting through different aspects. Among the established ACR TI-RADS ultrasound features, GPT-4o tended to detect the composition and the shape of thyroid nodules better than the other 3 characteristics.

To protect patients’ privacy and avoid possible pitfalls in clinical application of LLMs, all identification information was carefully removed. Similar to Wu’s work, since dialogues in LLMs were deemed relatively independent and could generate diverse answers, it was essential to evaluate the intra-LLM consistency of GPT-4o to validate the reproducibility of the application [22]. GPT-4o only reached fair to moderate agreement during 3 attempts of analyzing original thyroid sonography images in a zero-shot setting, with a relatively low κ coefficient and AC1 value. We then tried asking GPT-4o to mark several nodules in images and got mostly incorrect answers with noticeable extra-thyroidal marking, accounting for its low consistency and accuracy, as different regions were regarded as lesions among attempts. The obvious increase in GPT-4o’s consistency to judge marked nodules further supported this, demonstrating the relevance of the segmentation method. In a few-shot setting, the κ coefficient and the AC1 value were even higher, suggesting better agreement resulting from a steadier way for analysis established in GPT-4o’s algorithm based on provided examples with definite features and categories described. However, Krishna *et al*. held that a κ coefficient less than 0.91 or failure to reach almost perfect agreement was considered suboptimal because of identical questions and prompts [36, 37]. In the present study, although analysis of marked lesions achieved at least substantial agreement, only the few-shot marked group reached an AC1 value of 0.823 (almost perfect agreement), indicating that improvement in the reproducibility of LLMs was still needed for clinical decision-making assistance.

As for the accuracy, GPT-4o correctly identified the ACR TR category of a few nodules in original unmarked images, possibly owing to its inability to find the nodules in images as well. The accuracy significantly increased when the nodules were marked, but it still remained unsatisfactory and was close to previous research on image analysis by zero-shot setting of multimodal LLMs [37-39]. In a few-shot setting with 3 examples provided, the accuracy improved greatly. We hypothesized that the ability of GPT-4o to detect detailed radiologic features was inherently weak and examples were necessary for definition, so we gained some insights in the following analysis. We found GPT-4o tended to judge certain ultrasound features in an “all or none” manner when confronting signs presented in the 3 examples, when we calculated its precision and recall, so it was noteworthy that answers could be possibly homogenized with insufficient training examples in a few-shot setting. Unlike a convolutional neural network trained with large-scale radiologic data to solve specific problems, multimodal LLMs were pre-tuned for their application under many circumstances with higher interpretability. According to recent studies [40-42], the chain-of-thought strategy boosted the performance of LLMs in specialized medical fields, which could be practical for better adjustments. Simultaneously, retrieval-augmented generation and adaptation were conducive to the improvement of multimodal LLMs to generate correct judgments in clinical applications [14, 43-46].

To explain GPT-4o’s accuracy in analyzing real-world sonography, we investigated its accuracy in detecting detailed ACR TI-RADS ultrasound features. The accuracy to detect composition, shape, and margin of marked nodules was higher than that of unmarked ones, which emphasized that finding the nodule was the first step for improvement of LLMs, as only a correctly recognized nodule region provided a correct analysis of its outline. Consequently, combining multimodal LLMs with deep-learning-based segmentation models may help clinical applications. The few-shot setting significantly increased its accuracy to detect echogenicity, margin, and echogenic foci, which suggested that examples were pivotal for LLMs to analyze subtle features like localized brightness comparison. Echogenicity and calcifications were still difficult for GPT-4o because these features were mainly determined by analyzing the brightness distribution in images with various gains. By and large, GPT-4o did significantly better in detecting the composition and shape of the nodules.

Finally, we compared the performance of GPT-4o with young junior radiologists, and the results revealed a comparable accuracy, of GPT-4o improved by a few-shot setting and segmentation methods. Improved GPT-4o (few-shot marked group) performed even better in detecting the margin of thyroid nodules, underscoring the importance of segmentation. In the present study, we adopted an artificial segmentation approach as a proof-of-concept confirmation, but integration of deep-learning-based segmentation tools could be more effective in further simplifying clinical workflow and truly reducing radiologists’ burden [47-49]. Taking all these data into account, GPT-4o with few-shot setting and segmentation showed promising potential for primary report generation in the clinical ultrasound examination process.

## 5 STUDY LIMITATIONS

There were some limitations to this study. First, as a preliminary study to evaluate GPT-4o’s capacity for analyzing real-world sonography images for report generation facilitation, the present study was a single-center, retrospective study with a relatively small sample size of 120. Despite the promising estimated power for statistical tests, potential bias could still stem from ultrasound system variability or image quality factors, and multicenter studies with a large sample size should be needed to confirm the conclusion. Second, the proportion of TR5 nodules was excessive for the following 2 reasons, and possible bias might occur. Suspected malignancies were more common in our hospital as a tertiary transfer center because most patients came for surgery or ablation. Besides, most TR1 and TR2 nodules failed to match our inclusion criteria. Third, we did not investigate GPT-4o’s judgments upon thebenignormalignantnature of the nodules like Chen *et al.* [34] owing to the unavailability (FNA usually recom-mended for nodules above TR3) and some uncertain or false-negative results of FNA [50, 51]. It was reported that about 18% Bethesda II nodules, 25% Bethesda III nodules, and up to about 50% Bethesda IV nodules were malignant in recent studies [52-54]. Also, we tended to explore LLMs’ novel translational assistance in aiding report generation and improving ultrasound workflow rather than obtaining a definite diagnosis of thyroid nodules. Last but not least, GPT-4o could analyze video clips, but we did not attempt to investigate it yet. Further research on its performance to analyze video clips of dynamic examinations or contrast-enhanced ultrasound (CEUS) may be more meaningful for ultrasound diagnosis. Future studies can focus on multicenter confirmation of multimodal LLMs’ facilitating capacity, exploration of multi-modal LLMs’ capacity in analyzing multimodal ultrasound data, including CEUS video clips and elastography, compar-ison of different multimodal LLMs, and investigation of multi-modal LLMs’ capacity of interpreting cytological images of FNA.

## CONCLUSION

In conclusion, the performance of GPT-4o to analyze original real-world thyroid sonography images is insufficient, but it can be improved gradually by segmentation methods and a few-shot setting, which finally achieves a comparable level to junior radiologists with 1-3 years of experience in this preliminary study. Improved GPT-4o can potentially be conducive to real-world generation of preliminary radiological reports and improved ultrasound scan workflow, while multicenter validation is still needed.

## Figures and Tables

**Table 1:** Demographic and imaging characteristics of thyroid nodules.

**Characteristics**	**Value ^a^**	***p-*value ^b^**	**Characteristics**	**Value ^a^**	***p*-value ^b^**
Position		0.926	TR Category		0.874
Left lobe	54 (45.00%)		TR1	10 (8.33%)	
zero-shot	37 (46.25%)		zero-shot	6 (7.50%)	
few-shot	17 (42.50%)		few-shot	4 (10.00%)	
Right lobe	63 (52.50%)		TR2	15 (12.50%)	
zero-shot	41 (51.25%)		zero-shot	10 (12.50%)	
few-shot	22 (55.00%)		few-shot	5 (12.50%)	
Isthmus	3 (2.50%)		TR3	9 (7.50%)	
zero-shot	2 (2.50%)		zero-shot	5 (6.25%)	
few-shot	1 (2.50%)		few-shot	4 (10.00%)	
Size (cm)		0.209	TR4	9 (7.50%)	
Range	0.20-5.00		zero-shot	7 (8.75%)	
Mean	1.50±1.14		few-shot	2 (5.00%)	
zero-shot	1.41±1.07		TR5	77 (64.17%)	
few-shot	1.69±1.26		zero-shot	52 (65.00%)	
			few-shot	25 (62.50%)	

**Note:** ^a^ Data were expressed in the form of mean ± standard deviation or number (percentage). ^b^ Comparison: zero-shot vs few-shot. No statistically significant difference was found.

**Table 2:** Intra-LLM consistency of GPT-4o to analyze thyroid sonography.

Group	Attempts	Cohen κ ^a^	Fleiss κ ^a^	AC1 value ^a^
Zero-shot unmarked group	Attempt 1 vs 2	0.331 [0.178,0.484]	0.327 [0.249,0.406]	0.466 [0.367,0.564]
	Attempt 1 vs 3	0.353 [0.194,0.512]		
	Attempt 2 vs 3	0.310 [0.151,0.469]		
Zero-shot marked group	Attempt 1 vs 2	0.733 [0.604,0.862]	0.695 [0.608,0.783]	0.778 [0.696,0.860]
	Attempt 1 vs 3	0.640 [0.495,0.785]		
	Attempt 2 vs 3	0.715 [0.580,0.850]		
Few-shot marked group	Attempt 1 vs 2	0.798 [0.647,0.949]	0.755 [0.651,0.860]	0.823 [0.711,0.934]
	Attempt 1 vs 3	0.714 [0.547,0.881]		
	Attempt 2 vs 3	0.755 [0.592,0.918]		

**Note:** ^a^ Data were expressed in the form of value [95%CI].

**Table 3:** Accuracy of GPT-4o in analysis of TR category of thyroid nodules.

**Group**	**Attempt**	**Accuracy ^a^**	***p*-value ^b^**	**Mean accuracy ^a^**	***p*-value ^c^**
Zero-shot unmarked group	Attempt 1	17.50% (14/80) [8.99%,26.01%]	0.783	18.75% (45/240) [13.78%,23.72%]	<0.001*
	Attempt 2	21.25% (17/80) [12.09%,30.41%]			
	Attempt 3	17.50% (14/80) [8.99%,26.01%]			
Zero-shot marked group	Attempt 1	45.00% (36/80) [33.86%,56.14%]	0.816	42.50% (102/240) [36.20%,48.80%]	
	Attempt 2	40.00% (32/80) [29.03%,50.97%]			<0.001*
	Attempt 3	42.50% (34/80) [31.43%,53.57%]			
Few-shot marked group	Attempt 1	80.00% (32/40) [67.04%,92.96%]	0.386	79.17% (95/120) [71.80%,86.54%]	
	Attempt 2	85.00% (34/40) [73.43%,96.57%]			
	Attempt 3	72.50% (29/40) [58.04%,86.96%]			

**Note:** ^a^ Data were expressed in the form of percentage (correct cases/all cases) [95%CI]. ^b^ Comparison of accuracy among 3 attempts in zero-shot unmarked group, zero-shot marked group and few-shot marked group. ^c^ Comparison of mean accuracy: zero-shot unmarked group vs zero-shot marked group, zero-shot marked group vs few-shot marked group. * Statistically significant.

**Table 4:** Macro-average of Precision, Recall, and F1-score of GPT-4o to judge ACR TR categories and to identify ACR TI-RADS ultrasound features in 3 groups.

**Characteristics**	**Groups**	**Precision ^a^**	**Recall ^a^**	**F1-score ^a^**
TR category	Zero-shot unmarked group	0.264	0.209	0.233
	Zero-shot marked group	0.453	0.392	0.420
	Few-shot marked group	0.638	0.617	0.627
Composition	Zero-shot unmarked group	0.417	0.433	0.425
	Zero-shot marked group	0.552	0.458	0.501
	Few-shot marked group	0.771	0.687	0.727
Echogenicity	Zero-shot unmarked group	0.391	0.290	0.333
	Zero-shot marked group	0.381	0.327	0.352
	Few-shot marked group	0.542	0.525	0.533
Shape	Zero-shot unmarked group	0.426	0.467	0.446
	Zero-shot marked group	0.727	0.678	0.701
	Few-shot marked group	0.801	0.798	0.799
Margin	Zero-shot unmarked group	0.363	0.354	0.358
	Zero-shot marked group	0.468	0.506	0.486
	Few-shot marked group	0.588	0.619	0.603
Calcification	Zero-shot unmarked group	0.310	0.280	0.294
	Zero-shot marked group	0.326	0.311	0.318
	Few-shot marked group	0.396	0.378	0.387

**Note:** ^a^ Data presented were the macro-average of precision, recall, and F1-score respectively in each groups.

**Table 5:** Accuracy of GPT-4o in analysis of ultrasound features of thyroid nodules.

**Features**	**Group**	**Mean Accuracy ^a^**	***p*-value ^b^**
Composition	Zero-shot unmarked group	67.08% (161/240) [61.10%,73.07%]	<0.01*
	Zero-shot marked group	80.42% (193/240) [75.36%,85.47%]	/
	Few-shot marked group	88.33% (106/120) [82.51%,94.16%]	0.059
Echogenicity	Zero-shot unmarked group	51.25% (123/240) [44.88%,57.62%]	0.170
	Zero-shot marked group	57.50% (138/240) [51.20%,63.80%]	/
	Few-shot marked group	78.33% (94/120) [70.86%,85.81%]	<0.01*
Shape	Zero-shot unmarked group	60.83% (146/240) [54.61%,67.05%]	<0.01*
	Zero-shot marked group	75.83% (182/240) [70.38%,81.29%]	/
	Few-shot marked group	80.00% (96/120) [72.74%,87.26%]	0.375
Margin	Zero-shot unmarked group	37.92% (91/240) [31.73%,44.10%]	<0.01*
	Zero-shot marked group	69.58% (167/240) [63.72%,75.45%]	/
	Few-shot marked group	89.17% (107/120) [83.53%,94.81%]	<0.01*
Echogenic foci	Zero-shot unmarked group	47.08% (113/240) [40.72%,53.44%]	0.236
	Zero-shot marked group	52.50% (126/240) [46.14%,58.86%]	/
	Few-shot marked group	73.33% (88/120) [65.31%,81.36%]	<0.01*

**Note:** ^a^ Data were expressed in the form of mean percentage (correct cases/ all cases) [95%CI]. ^b^ Comparison of mean accuracy: zero-shot unmarked group vs zero-shot marked group, zero-shot marked group vs few-shot marked group. * Statistically significant.

## Data Availability

The data of current study are available from corresponding authors, [M.X] and [X.Z], on a reasonable request.

## LIST OF ABBREVIATIONS

95%CI: 95% confidence interval
ACR TI-RADS: American College of Radiology Thyroid Imaging Reporting and Data System
AI: Artificial Intelligence
ChatGPT: Generative pre-trained transformer
CEUS: Contrast-enhanced ultrasound
CT: Computed tomography
FNA: Fine-needle aspiration
GPT-4o: GPT-4 Omni
LLM: Large language model

## STANDARDS OF REPORTING

STROBE guidelines were followed.
