# Supplementary material for: Evaluating Consistency and Accuracy of GPT-4 Omni to Analyze Thyroid Ultrasound Features and ACR TR Categories to Aid Report Generation
Source: Curr Med Imaging. 2026 Mar 12;22:e15734056437835. doi: 10.2174/0115734056437835260214191655 (PMC13483112; doi:10.2174/0115734056437835260214191655)
Supplement: Supplementary file 1 [file CMIM-22-E15734056437835_SD1.pdf]

1875-6603/26

Send Orders for Reprints to reprints@benthamscience.net

BENTHAM SCIENCE

CrossMark

Current Medical Imaging

Content list available at: https://benthamscience.com/journals/cmir

Supplementary Material

Evaluating Consistency and Accuracy of GPT-4 Omni to Analyze Thyroid Ultrasound Features and ACR TR Categories to Aid Report Generation

Zebang Yang[1,#], Tongyi Huang[1,#], Lei Huang[1,#], Jiaqian Yao[1], Lin Jiang[1], Wenxin Wu[1], Xiaoyan Xie[1], Ming Xu[1,*] and Xiaoer Zhang[1,*]

[1]Department of Medical Ultrasonics, Institution of Diagnostic and Interventional Ultrasound, The First Affiliated Hospital of Sun Yat-sen University, No.58 Zhongshan Er Road, Guangzhou 510080, Guangzhou, People's Republic of China

Supplementary Material: Format of Prompts

Introduction Prompts (input at the beginning of each dialogue):

Do you know the ACR TI-RADS (American College of Radiology Thyroid Imaging Reporting and Data System) category of thyroid nodules? It evaluates the component. the echogenicity, the shape, the margin, and hyperechoic foci of the thyroid nodules and then a score for prediction of malignancy will be calculated.

Prompts for unmarked nodules:

Here are two images of ultrasound scan of the same thyroid nodule (Patient No.X). The first image shows the cross section of the nodule while the second image shows its longitudinal section. Please judge the TR category of the nodule according to the ACR TI-RADS score.

Prompts for marked nodules:

Here are two images of ultrasound scan of the same thyroid nodule (Patient No.X). The first image shows the cross section of the nodule while the second image shows its longitudinal section. The nodule is marked in the red circle. Please judge the TR category of the nodule according to the ACR TI-RADS score.

Prompts in the few-shot setting (introducing examples):

Here is the first/second/third example. These are images of ultrasound scan of a thyroid nodule marked in the red circle (Example No.X). The first image shows the cross section of the nodule while the second image shows its longitudinal section. We can see that the nodule is predominantly cystic / mixed cystic and solid/ predominantly solid (X points), …echoic (X points, compared to surrounding thyroid tissue), wider-than-tall/ taller-than-wide in cross section (X points), with … margin (X points) and … echogenic foci (X points), which accounts for X points in total and falls into TRX according to ACR TI-RADS categories.

(Changeable parts marked in red color.)

© 2026 The Author(s). Published by Bentham Science Publisher.

This is an open access article distributed under the terms of the Creative Commons Attribution 4.0 International Public License (CC-BY 4.0), a copy of which is available at: https://creativecommons.org/licenses/by/4.0/legalcode. This license permits unrestricted use, distribution, and reproduction in any medium, provided the original author and source are credited.

HOW TO CITE:
Yang Z, Huang T, Huang L, Yao J, Jiang L, Wu W, Xie X, Xu M, Zhang X. Evaluating Consistency and Accuracy of GPT-4 Omni to Analyze Thyroid Ultrasound Features and ACR TR Categories to Aid Report Generation. Curr Med Imaging, 2026; 22: e15734056437835.
http://dx.doi.org/10.2174/0115734056437835260214191655
